# A Rapid Antimicrobial Resistance Diagnostic Platform for Staphylococcus aureus Using Recombinase Polymerase Amplification

**DOI:** 10.1128/spectrum.04476-22

**Published:** 2023-03-28

**Authors:** Chuangxing Lin, Yongmei Zeng, Zhihong Zhu, Jiayu Liao, Tiandan Yang, Yaqun Liu, Huagui Wei, Jiamin Li, Jibin Ma, Xiaoqing Wu, Guangyu Lin, Liyun Lin, Liying Chen, Huiying Huang, Weizhong Chen, Junli Wang, Feiqiu Wen, Min Lin

**Affiliations:** a Department of Pediatrics, Second Affiliated Hospital of Shantou University Medical College, Shantou, Guangdong, China; b Department of Pediatric Hematology and Oncology, Shenzhen Children's Hospital, China Medical University, Shenzhen, Guangdong, China; c Department of Endocrinology, Second Affiliated Hospital of Shantou University Medical College, Shantou, Guangdong, China; d School of Food Engineering and Biotechnology, Hanshan Normal University, Chaozhou, Guangdong, China; e School of Laboratory Medicine, Youjiang Medical University for Nationalities, Baise, Guangxi, China; f Guangxi Medical and Health Key Discipline Construction Project of the Affiliated Hospital of Youjiang Medical University for Nationalities, Baise, Guangxi, China; g Department of Medical Laboratory, Chaozhou People’s Hospital Affiliated to Shantou University Medical College, Chaozhou, Guangdong, China; University of Cincinnati

**Keywords:** *Staphylococcus aureus*, antimicrobial resistance, recombinase polymerase amplification, rapid detection

## Abstract

Antimicrobial resistance (AMR) has posed a global threat to public health. The Staphylococcus aureus strains have especially developed AMR to practically all antimicrobial medications. There is an unmet need for rapid and accurate detection of the S. aureus AMR. In this study, we developed two versions of recombinase polymerase amplification (RPA), the fluorescent signal monitoring and lateral flow dipstick, for detecting the clinically relevant AMR genes retained by S. aureus isolates and simultaneously identifying such isolates at the species level. The sensitivity and specificity were validated with clinical samples. Our results showed that this RPA tool was able to detect antibiotic resistance for all the 54 collected S. aureus isolates with high sensitivity, specificity, and accuracy (all higher than 92%). Moreover, results of the RPA tool are 100% consistent with that of PCR. In sum, we successfully developed a rapid and accurate AMR diagnostic platform for S. aureus. The RPA might be used as an effective diagnostic test in clinical microbiology laboratories to improve the design and application of antibiotic therapy.

**IMPORTANCE** Staphylococcus aureus is a species of Staphylococcus and belongs to Gram-positive. Meanwhile, S. aureus remains one of the most common nosocomial and community-acquired infections, causing blood flow, skin, soft tissue, and lower respiratory tract infections. The identification of the particular *nuc* gene and the other eight genes of drug-resistant S. aureus can reliably and quickly diagnose the illness, allowing doctors to prescribe treatment regimens sooner. The detection target in this work is a particular gene of S. aureus, and a POCT is built to simultaneously recognize S. aureus and analyze genes representing four common antibiotic families.

We developed and assessed a rapid and on-site diagnostic platform for the specific and sensitive detection of S. aureus. This method allows the determination of S. aureus infection and 10 different AMR genes representing four different families of antibiotics within 40 min. It was easily adaptable in low-resource circumstances and professional-lacking circumstances. It should be supported in overcoming the continuous difficulty of drug-resistant S. aureus infections, which is a shortage of diagnostic tools that can swiftly detect infectious bacteria and numerous antibiotic resistance indicators.

## INTRODUCTION

Antimicrobial resistance (AMR) is the capacity of microorganisms, including bacteria, viruses, parasites, and fungi, to thwart the effects of antimicrobial treatments, such as antibiotics, antiviral medications, antimalarial medications, or antifungal medications. In recent years, the abuse and misuse of antimicrobials between humans and animals has continuously increased the number and types of drug-resistant microorganisms, which has become one of the biggest threats to global public health security (1). More than 700,000 people die from it each year, and by 2050 (2, 3) that figure might reach 10 million. Drug-resistant pathogen growth is mostly fueled by the abuse and overuse of antimicrobial drugs (4). If the dosage of antimicrobial drugs is insufficient or the wrong kind of antibiotics are used, the pathogen may develop AMR. Minimizing the impact of AMR is a global priority (5).

Staphylococcus aureus is a significant pathogen that causes a number of nosocomial and community-related illnesses that have high morbidity and death rates (6, 7). S. aureus is quite plastic and may respond to a variety of environmental factors (8). S. aureus strains have developed resistance mechanisms to practically all antimicrobial medications used in therapy, including beta-lactams, glycopeptides, and tetracyclines (7). Therefore, to swiftly discover illnesses, bacteria, and their associated antimicrobial resistance profiles, a sensitive and effective diagnostic approach is essential. This is necessary for clinical diagnosis to provide targeted antimicrobial treatment and lower the risk of death.

Current gold standard procedures for identifying AMR of S. aureus include classic culture-biochemical and antimicrobial susceptibility-testing methods (9). These methods are bacterial growth tests in the presence of antibiotics that allow the pathogen’s susceptibility or resistance to a given antibiotic to be determined. These tests are often time-consuming, and the results generally take 20 h to 72 h (7), thereby delaying treatment. Furthermore, bacterial strains with AMR genes may exhibit antibiotic sensitivity owing to a lack of gene expression or subpar development, which may lead to misleading negative findings. Molecular diagnostic tests, such as PCR-based technologies (10), matrix-assisted laser desorption/ionization time-of-flight mass spectrometry (MALDI-TOF MS) (11), and next-generation sequencing (12), can be performed directly on clinical samples, providing earlier information about the clinical strain's resistance profile. However, they are complicated, require specific skills and knowledge, or depend on pricey equipment, making them unsuitable for usage in rural or primary care, especially in resource-limited nations.

Recombinase polymerase amplification (RPA) is a new isothermal amplification method that might be a viable alternative to PCR (13). RPA amplification products can be identified using agarose gel electrophoresis (AGE), fluorescent signal monitoring (FSM), and lateral flow dipstick (LFD) (14, 15). When identifying materials with a high impurity level, the RPA has relatively few problems. It is made possible by the diagnostic demands for quick, on-site, sensitive, and portable tests (13, 14, 16). Here, a rapid antimicrobial resistance diagnostic platform for S. aureus using RPA was developed and assessed. This method allows the determination of S. aureus infection and nine different AMR genes representing four different families of antibiotics within 40 to 50 min. Under optimal conditions, the platform was evaluated methodologically with culture-based antimicrobial susceptibility-testing and PCR. To meet the requirements of different scenarios, the results can be interpreted in two different forms, including FSM and LFD. It will offer a powerful tool for future surveillance of antimicrobial resistance in patients in the field (2).

## RESULTS

### Overall workflow for the AMR diagnostic platform.

**FIG 1:**
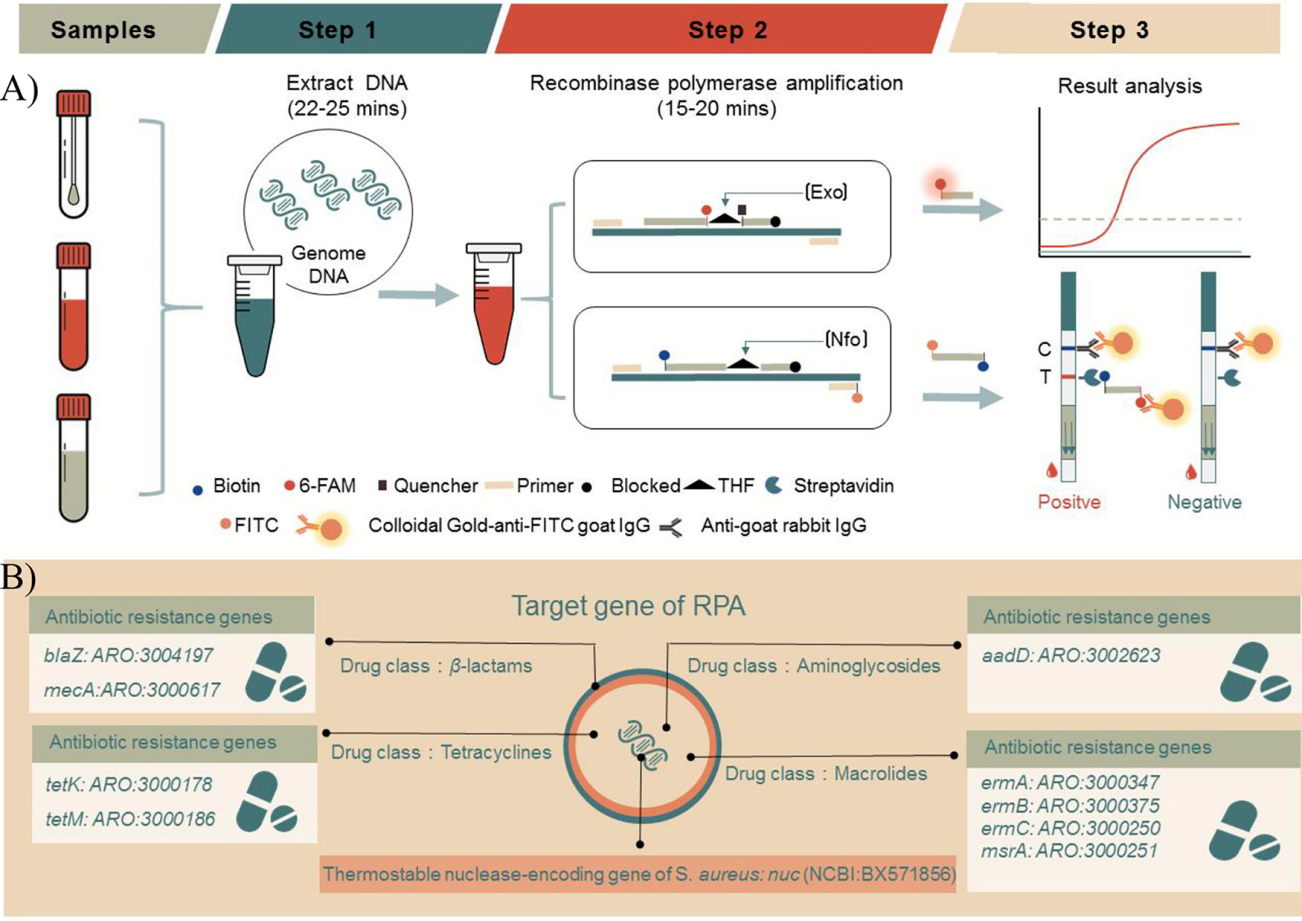
Workflow for the AMR diagnostic platform. (A) Timeline and workflow for the AMR diagnostic platform using clinical samples. Samples are collected and extracted from clinical patients (swabs, blood, secretion, and others). DNA was amplified at one temperature, removing the need for a thermocycler. The final DNA product was identified using RPA-LFD and RPA-FSM. (B) The target genes of the RPAs: thermostable nuclease gene (nuc) and nine AMR genes of S. aureus.

The AMR diagnostic platform's process, from sample collection through interpretation of the final results, is shown in Fig. 1A. DNA was extracted from clinical samples collected from patients, followed by recombinase polymerase amplification (RPA) at 37 to 39°C. First, unique genomic regions of the *nuc* gene were amplified for the identification of S. aureus infection. Second, the drug resistance genotypes of S. aureus were detected by targeted amplification of serious AMR genes representing four different families of antibiotics, such as *blaZ* and *mecA* (Fig. 1B). To better apply in various scenarios, we designed the RPA-FSM version and RPA-LFD version. For low-resource locations, RPA-Nfo primer and probe sets combined with LFD could rapidly provide visible results without high-cost equipment. For basic medical constitutions that professionals lack, the FSM version using the RPA-Exo probe could simply realize quantitative analysis.

### Primer and probe screening.

**FIG 2:**
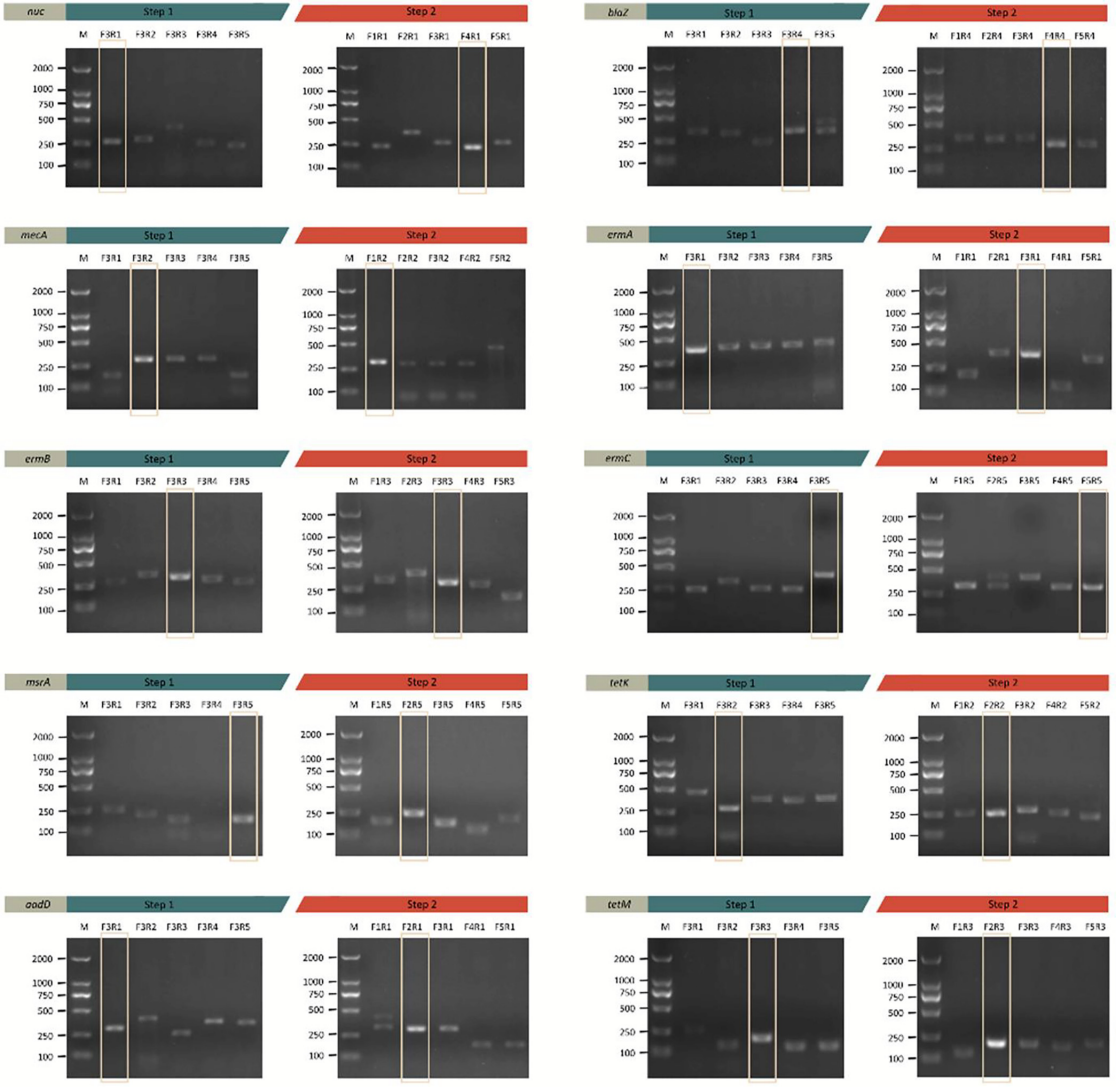
Screening all reverse primers against a single forwards primer, picking the best reverse primer and then using it to screen all the forwards primers, the good primer pairs can be found in 16 to 20 reactions. For the *nuc* gene, all reverse primers were initially matched by the forwards primer F3. The F3R1 band was discovered to be the most positive and clear. The best reverse primer, as a result, was R1. Then, all of the forwards primers were matched using R1 primers. The best primer pair, according to the AGE results, was F4R1. The other primer pairs for the same strategy were tested.

**FIG 3:**
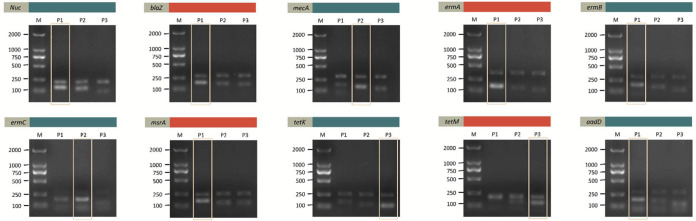
The Prode screening strategy of all genes. Taking the *nuc* as an example, three probes were separately paired up to F4R1 (the best primer pair). According to Fig. 3, the probe P1-F4R1 combination could amplify two positive and clear bands. Based on the aforementioned findings, the P1-F4R1 combination was approved for use in the experiments that followed. All optimum probe-primer combinations targeted to the other genes were confirmed using the same strategy.

To target each gene, we designed five pairs of primers and three probes for screening. The above-mentioned screening strategy was used for screening all candidate primers and probes. Taking the “*nuc* gene” as an example, we designed primers after comparing the *nuc* gene (ATCC number) of various bacterial strains. As shown in Fig. 2, in step 1, forwards primer F3 was used to match all reverse primers. It was found that the F3R1 band (250 bp) was the most positive and clear. Thus, the R1 primer was determined to be the best reverse primer. In step 2, the R1 primer was used to match all the forwards primers. The AGE results showed that F4R1 was the best primer pair. Moreover, three paired probes were paired up to F4R1 separately. As shown in Fig. 3, the probe P1-F4R1 combination amplified two positive and clear bands. Based on the above results, the P1-F4R1 combination was confirmed for use in the following experiments. For the same strategy, all optimal probe-primer combinations targeting the *nuc* gene and nine AMR genes were confirmed (Table 1).

**TABLE 1:** Nucleotide sequences of the primers and probes of the AMR diagnostic platform

Target gene	Primers and probes	Sequence (5′–3′)	Primers product size (bp)
(A) nucleotide sequences of the primers and probes of RPA-FSM		
*Nuc*	*nuc*-exo-F4	AACAGATAATGGCGTAAATAGAAGTGGTTC	225
	*nuc*-exo-R1	CACTTGCTTCAGGACCATATTTCTCTACAC	
	*nuc*-exo-P01	AAAGAACCTGCGACATTAATTAAAGCGAT(FAM-dT)(THF)A(BHQ1-dT)GGTGATACTGTTA-C3-spacer	
*blaZ*	*blaZ*-exo-F4	TCGCAAATGGAAAATTAAGCAAAGAAAACA	283
	*blaZ*-exo-R4	TTCATTACACTCTTGGCGGTTTCACTTATC	
	*blaZ*-exo-P01	AAGGTTGCTGATAAAAGTGGTCAAGCAA(FAM-dT)A(THF)CA(BHQ1-dT)ATGCTTCTAGAA-C3-spacer	
*mecA*	*mecA*-exo-F1	CCTGTTTGAGGGTGGATAGCAGTACCTGAGCC	305
	*mecA*-exo-R2	GGTTATGTTGGTCCCATTAACTCTGAAGAA	
	*mecA*-exo-P01	CTATTATCGTCAACGATTGTGACACGA(FAM-dT)AG(THF)CA(BHQ1-dT)CTTCATGTTGGA-C3-spacer	
*ermA*	*ermA*-exo-F3	GCTAGTCAAAATGAGTCGATCAGTTACTGCT	373
	*ermA*-exo-R1	AGAGTCTACACTTGGCTTAGGATGAAAATA	
	*ermA*-exo-P01	TTTGAAAGTCAGGCTAAATATAGCTATCT(FAM-dT)(THF)(BHQ1-dT)CGTTGAGAAGGGAT-C3-spacer	
er*mB*	*ermB*-exo-F3	TTTTGAAAGCCATGCGTCTGACATCTATCT	312
	*ermB*-exo-R3	GCGTGTTTCATTGCTTGATGAAACTGATTT	
	*ermB*-exo-P01	TGCTTAAGCTGCCAGCGGAATGCTTTCA(FAM-dT)C(THF)(BHQ1-dT)AAACCAAAAGTAAA-C3-spacer	
*ermC*	*ermC*-exo-F5	TCGTGGAATACGGGTTTGCTAAAAGATTAT	302
	*ermC*-exo-R5	ATTGTTTAAATCGTCAATTCCTGCATGTTT	
	*ermC*-exo-P02	CCTAAACCTAAAGTGAATAGCTCACTTA(FAM-dT)C(THF)GA(BHQ1-dT)TAAATAGAAAAA-C3-spacer	
*msrA*	*msrA*-exo-F2	AAGTCAAAAACTGCTAACACAAGTACGATTC	251
	*msrA*-exo-R5	TAGCTCTACTGAATGATTCTGATGAATCCGT	
	*msrA*-exo-P01	GGTGTAGGTAAGACAACTTTACTTGAAGC(FAM-dT)(THF)(BHQ1-dT)TTACCACCAAATAG-C3-spacer	
*tetK*	*tetK*-exo-F2	GAATATAATGTGCTATTATTCCCCCTATTGAA	257
	*tetK*-exo-R2	GTTAATTATTGGTATTAGTTTGAGCTGTCTTG	
	*tetK*-exo-P03	AAGGGAATGCAGCAGATCCTACTCCTTG(FAM-dT)A(THF)(BHQ1-dT)AACCTACCAAAAAT-C3-spacer	
tetM	*tetM*-exo-F2	ACCAAAATGGAATTGATTTATCAACGGTTT	211
	*tetM*-exo-R3	TCGAGTTCCAATGCTTCTAATGATTTACCG	
	*tetM*-exo-P03	AACAGAAGGTAGAACTGTATCCTAATATG(FAM-dT)(THF)(BHQ1-dT)GTGACGAACTTTAC-C3-spacer	
aadD	*aadD-*exo*-F2*	GGCTATTGGTGTTTATGGCTCTCTTGGTCGT	295
	*aadD-*exo*-R1*	CGTTTGGGCTTCTACCGATTTAGCAGTTTGA	
	*aadD*-exo*-P01*	CAACCGGTGAGTGGAAGGTGGAAGTGAAT(FAM-dT)(THF)(BHQ1-dT)GATAGCGAAGAGAT-C3-spacer	
(B) Nucleotide sequences of the primers and probes of RPA-LFD		
*Nuc*	*nuc*-nfo-F4	AACAGATAATGGCGTAAATAGAAGTGGTTC	225
	*nuc*-nfo-R1	FITC-CACTTGCTTCAGGACCATATTTCTCTACAC	
	*nuc*-nfo-P01	Biotin-AAAGAACCTGCGACATTAATTAAAGCGATT(THF)ATGGTGATACTGTTA-C3-spacer	
*blaZ*	*blaZ*-nfo-F4	TCGCAAATGGAAAATTAAGCAAAGAAAACA	283
	*blaZ*nfo-R4	FITC-TTCATTACACTCTTGGCGGTTTCACTTATC	
	*blaZ*-nfo-P01	Biotin-AAGGTTGCTGATAAAAGTGGTCAAGCAATA(THF)CATATGCTTCTAGAA-C3-spacer	
*mecA*	*mecA*-nfo-F1	CCTGTTTGAGGGTGGATAGCAGTACCTGAGCC	305
	*mecA*-nfo-R2	FITC-GGTTATGTTGGTCCCATTAACTCTGAAGAA	
	*mecA*-nfo-P01	Biotin-CTATTATCGTCAACGATTGTGACACGATAG(THF)CATCTTCATGTTGGA-C3-spacer	
*ermA*	*ermA*-nfo-F3	GCTAGTCAAAATGAGTCGATCAGTTACTGCT	373
	*ermA*-nfo-R1	FITC-AGAGTCTACACTTGGCTTAGGATGAAAATA	
	*ermA*-nfo-P01	Biotin-TTTGAAAGTCAGGCTAAATATAGCTATCTT(THF)TCGTTGAGAAGGGAT-C3-spacer	
*ermB*	*ermB*-nfo-F3	TTTTGAAAGCCATGCGTCTGACATCTATCT	312
	*ermB*-nfo-R3	FITC-GCGTGTTTCATTGCTTGATGAAACTGATTT	
	*ermB*-nfo-P01	Biotin-TGCTTAAGCTGCCAGCGGAATGCTTTCATC(THF)TAAACCAAAAGTAAA-C3-spacer	
er*mC*	*ermC*-exo-F5	TCGTGGAATACGGGTTTGCTAAAAGATTAT	302
	*ermC*-nfo-R5	FITC-ATTGTTTAAATCGTCAATTCCTGCATGTTT	
	*ermC*-nfo-P02	Biotin-CCTAAACCTAAAGTGAATAGCTCACTTATC(THF)GATTAAATAGAAAAA-C3-spacer	
*msrA*	*msrA*-nfo-F2	AAGTCAAAAACTGCTAACACAAGTACGATTC	251
	*msrA*-nfo-R5	FITC-TAGCTCTACTGAATGATTCTGATGAATCCGT	
	*msrA*-nfo-P01	Biotin-GGTGTAGGTAAGACAACTTTACTTGAAGCT(THF)TTTACCACCAAATAG-C3-spacer	
*tetK*	*tetK*-nfo-F2	GAATATAATGTGCTATTATTCCCCCTATTGAA	257
	*tetK*-nfo-R2	FITC-GTTAATTATTGGTATTAGTTTGAGCTGTCTTG	
	*tetK*-nfo-P03	Biotin-AAGGGAATGCAGCAGATCCTACTCCTTGTA(THF)TAACCTACCAAAAAT-C3-spacer	
*tetM*	*tetM*-nfo-F2	ACCAAAATGGAATTGATTTATCAACGGTTT	211
	*tetM*-nfo-R3	FITC-TCGAGTTCCAATGCTTCTAATGATTTACCG	
	*tetM*-nfo-P03	Biotin-AACAGAAGGTAGAACTGTATCCTAATATGT(THF)TGTGACGAACTTTAC-C3-spacer	
*aadD*	*aadD-*nfo*-F2*	GGCTATTGGTGTTTATGGCTCTCTTGGTCGT	295
	*aadD-*nfo*-R1*	FITC-CGTTTGGGCTTCTACCGATTTAGCAGTTTGA	
	*aadD-*nfo*-P01*	Biotin-CAACCGGTGAGTGGAAGGTGGAAGTGAATT(THF)TGATAGCGAAGAGAT-C3-spacer	

### Optimization of the RPA method.

The AMR diagnostic platform was further optimized. The optimal reaction system of every gene, including the concentration of primers, probes, temperature, reaction time, and MgAo, is shown in Table S1. Various parameters are slightly different in the RPA-FSM version and RPA-LFD version. For example, the RPA-FSM displayed an optimization reaction temperature of 39°C at 20 min; however, the RPA-LFD displayed an optimization result of 37°C at 15 min.

### Diagnostic specificity and sensitivity testing.

**FIG 4:**
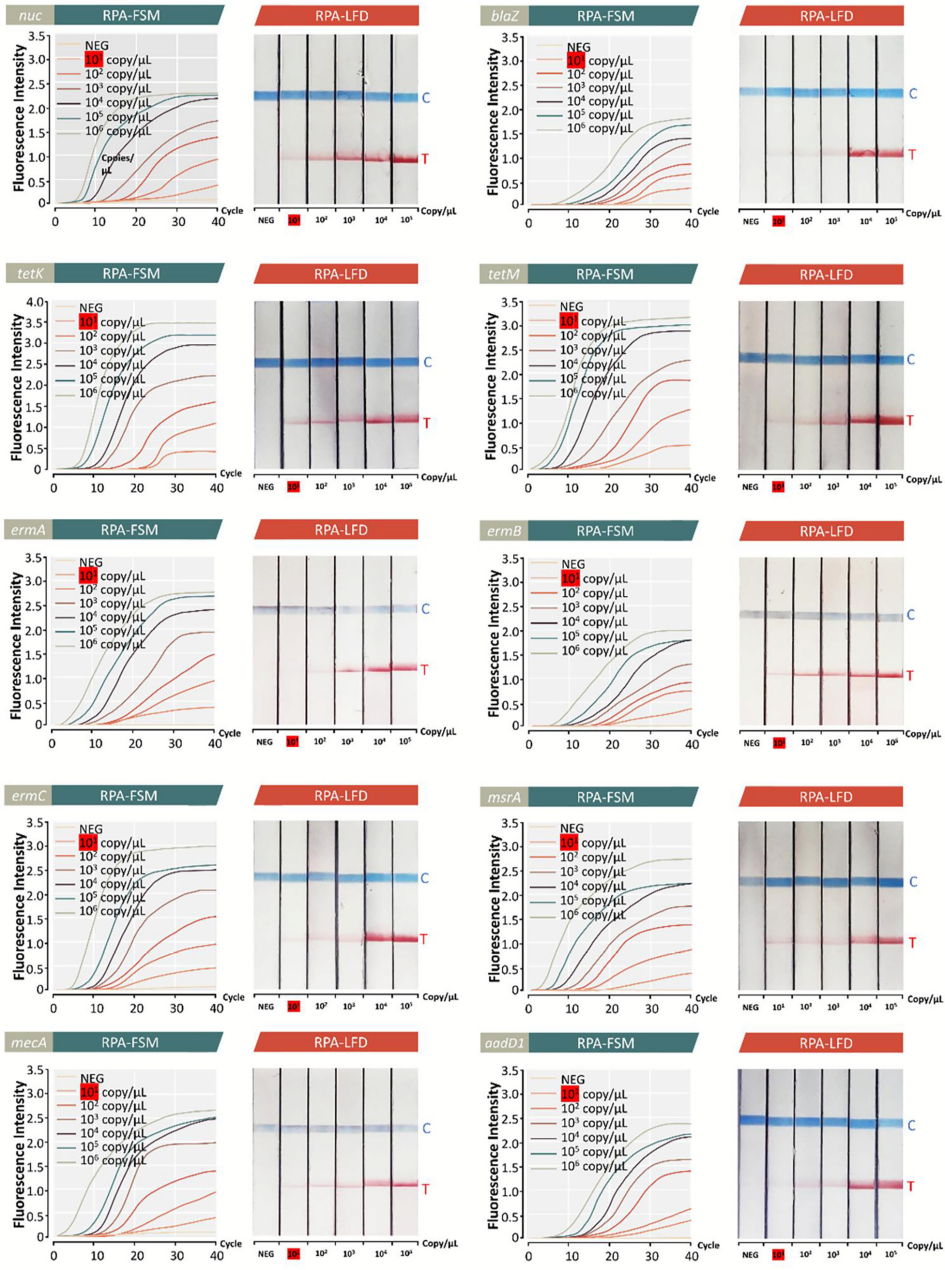
Each synthetic gene plasmid standard was used to determine the limit of detection (LOD) of the AMR diagnostic platform of S. aureus. Fig. 4 demonstrates that both the LOD of RPA-FSM and the LOD of RPA-LFD ranged from 10^1^ to 10^2^ copies/μL.

Reactions were carried out in conjunction with DNA from other bacteria in an attempt to demonstrate the specificity of the AMR detection platform under optimal conditions. There were no responses when other microorganisms were present, such as Klebsiella pneumoniae and Streptococcus pneumoniae, which were tested and detected by RPA-AGE (Fig. S1A), RPA-FSM (Fig. S1B), and RPA-LFD (Fig. S1C). Additionally, the plasmids of all the genes were mixed (10^5^ copies/μL), and the primer set targets of each gene were used to detect it. The outcomes demonstrated that these primers could not produce cross-reactions and could only amplify their own target genes (Fig. S2). To evaluate the limit of detection (LOD) of the AMR diagnostic platform of S. aureus, each synthetic gene plasmid standard was used. Fig. 4 demonstrates that both the LOD of RPA-FSM and the LOD of RPA-LFD ranged from 10^1^ to 10^2^ copies/μL.

### The clinical sensitivity and specificity for isolates.

To evaluate the diagnostic ability of this platform, 54 S. aureus isolates were collected from patients with suspected S. aureus infection. For the study, we first examined the samples for the presence of nine genes (*ermA*, *ermB*, *ermC*, *mecA*, *blaZ*, *tetK*, *tetM*, *msrA*, *aadD*) by our AMR diagnostic platform and PCR. Then, all samples were evaluated for resistance to penicillin, oxacillin, erythromycin, clindamycin, tetracycline, and gentamicin via cultivation-antimicrobial susceptibility. Compared with the culture results of the hospital diagnostic facilities, our AMR diagnostic tool was able to detect antibiotic resistance for 54 clinical strains with high sensitivity, specificity, and accuracy (Table 2 and S3). Our assay exhibited an accuracy of 92.59% for penicillin, 94.44% for oxacillin, 96.30% for erythromycin and clindamycin, 94.44% for tetracycline, and 96.30% for gentamicin. Additionally, all results indicated that our AMR diagnostic detection performance approached the performance of PCR, with a consistency rate of 100% (Table S2).

**TABLE 2:** Clinical sensitivity, specificity and accuracy of antimicrobial resistance genes in clinical sample

Antibiotic class	Antibiotic	Antimicrobial resistance genes	Sensitivity (%)	Specificity (%)	Accuracy (%)
β-lactams	oxacillin	*mecA*	95.45% (21/22)	93.75% (30/32)	94.44% (51/54)
	penicillin	*blaZ* and *mecA*	95.74% (45/47)	71.43% (5/7)	92.59% (50/54)
Macrolides	erythromycin	*ermA*, *ermB*, *ermC* and *msrA*	100% (25/25)	93.10% (27/29)	96.30% (52/54)
	clindamycin	*ermA*, *ermB*, *ermC* and *msrA*	96.15% (25/26)	92.86% (26/28)	94.44% (51/54)
	erythromycin and clindamycin	*ermA*, *ermB*, *ermC* and *msrA*	96.30% (26/27)	96.30% (26/27)	96.30% (52/54)
Tetracyclines	tetracycline	*tetK* and *tetM*	88.89% (16/18)	97.22% (35/36)	94.44% (51/54)
Aminoglycosides	gentamicin	*aadD*	100% (4/4)	96.00% (48/50)	96.30% (52/54)

## DISCUSSION

Multiple-antibiotic-resistant S. aureus strains are presently a major health care concern, as they are the causal agent of numerous nosocomial and community-acquired pathological disorders (17–19). For that reason, accurate and early identification of resistant isolates is a key objective of clinical microbiology, and point-of-care testing (POCT) has become a critical tool in laboratory operations (20, 21). POCT refers to a portable mobile detection system that is close to the test sample in a nonlaboratory environment and immediately reports the results (22–24). It has also become a critical detection technology for various specific pathogens (25–34). During the recent period, several studies have proven the exceptionally high capability of RPA for selectively recognizing microorganisms and genes of interest. This capacity has exposed RPA as a valuable POCT technique in clinical microbiology research (35–40). Several writers have previously proven the possibility of RPA or RPA incorporating various aptamer technologies, such as multicomponent nucleic acid enzymes (MNAzymes)-based techniques and CRISPR/Cas-based techniques (41, 42), for the identification of AMR S. aureus strains. Nevertheless, almost all RPA-based genotypic testing concentrates on determining both genes (*nuc* and *mecA*) associated with methicillin-resistant S. aureus (43–49). Our objective was to create a POCT for the simultaneous recognition of S. aureus and the analysis of genes representing four regularly used families of antibiotics.

Because S. aureus is one of the most frequently antibiotic-resistant bacteria, it often causes serious bloodstream, skin and soft tissue, and urinary tract infections in humans (6, 8, 19). Treatment with methicillin was regarded as a reliable therapeutic strategy for S. aureus infection until MRSA emerged and spread worldwide in past several decades (50). The development process of antibiotic-resistant S. aureus has gradually stabilized since the launch of the Global Antimicrobial Resistance Surveillance System (GLASS) in 2014. However, antibiotic-resistant S. aureus infections, especially MRSA, remain a major public health concern. Most of the 54 clinical isolates collected from different departments for this study showed antimicrobial resistance to at least one class of antibiotics. The resistance rate of S. aureus isolates to penicillin reached 87.0%, slightly lower than Massa et al. reported (51). In the meanwhile, S. aureus also had high resistance rate to methicillin (40.7%), clindamycin (48.1%), and erythromycin (46.3%). In contrast, vancomycin was the most effective antibiotic, with a 100% susceptibility, suggesting that vancomycin could be useful as a treatment for multiple antibiotic-resistant S. aureus infections. Recently, however, searchers from several countries have reported the occurrence of vancomycin-intermediate S. aureus (VISA) and vancomycin-resistant S. aureus (VRSA) successively (52). According to WHO guidelines, it would be more effective to detect resistance and use antibiotics reasonably and accurately, rather than develop new antibiotics (5). With the further investigation of S. aureus resistance and resistance mechanisms, resistance genes are playing an increasingly important role in S. aureus antimicrobial resistance, which makes good use of molecular diagnosis for the drug resistance prediction (44, 45, 48). In this study, we were devoted to detecting *nuc* gene and nine AMR genes by RPA to develop a rapid molecular diagnostic platform for S. aureus and its antimicrobial resistance. The AMR diagnostic performance has been found to have high sensitivity and specificity (Table 2). For the clinical samples in the study, there was 100% concordance between the AMR diagnostic testing and PCR results. In comparison to cultivation-antimicrobial susceptibility, our AMR diagnostic tool correctly recognized targets from clinical samples at concentrations as low as 10^5^ CFU/L and exhibited an accuracy of 92% to 97% for profiling pathogenic bacteria and predicting AMR phenotypes.

Several essential criteria are carefully evaluated, including speed, cost, simplicity of use, performance (sufficient sensitivity and specificity), and availability in our region. The RPA-LFD version and RPA-FSM version were designed to be used separately in low-resource circumstances and professional-lacking circumstances. Finally, the two versions for all genes (*nuc* gene and nine AMR genes) could be separately run under uniform conditions. Although the RPA-LFD version was more popular in the interpretation of results for its rapid visualization, the RPA-FSM version may drastically lower test costs. Currently, the commercial strip costs ⁓$4.50 per strip. As a result, the RPA-LFD version requires an additional ⁓$45 to complete the identification of 10 genes. Additionally, aerosol pollution caused by open cover detection could also limit RPA-LFD application. For these reasons, we believe that our RPA-FSM version will have more future application value.

In clinical practice, surveillance testing combined with source control strategies is an effective method for preventing the transmission of multiple antibiotic-resistant S. aureus. For example, limited nasal screening was carried out in high-risk patients (ICU patients and those outside the ICU with CVCs or midline catheters) or admission to high-risk settings (e.g., ICU). After collecting the samples with a nasal swab, the results should be available as soon as possible. Then, the patients colonized or infected with multiple-antibiotic-resistant S. aureus, particularly MRSA, were placed in private rooms and used contact precautions in inpatient acute care settings. These effective measures could drastically decrease the spread of MRSA among patients. Our AMR diagnostic performance could provide serious information within 40 to 50 min. It could shorten the detection time and be beneficial to the above implemented interventions.

### Conclusion.

In summary, a rapid and on-site diagnostic platform for the specific and sensitive detection of S. aureus was developed and assessed. This method allows the determination of S. aureus infection and nine different AMR genes representing four different families of antibiotics within 40 to 50 min. It was easily adaptable in low-resource circumstances and professional-lacking circumstances. It should be supported in overcoming the continuous difficulty of drug-resistant S. aureus infections, which is a shortage of diagnostic tools that can swiftly detect infectious bacteria and numerous antibiotic resistance indicators.

## MATERIALS AND METHODS

### Ethics statement.

This study was approved by the Medical Ethics Committee of the Second Affiliated Hospital of Shantou University Medical College (permit number: 2020-31). The clinical isolates were gathered between 2020 and 2021. All samples were taken with written authorization.

### Bacterial strain and DNA extraction.

A total of 16 reference bacterial strains of S. aureus (ATCC-25923), Staphylococcus hominis (ATCC-27844), Staphylococcus haemolyticus (ATCC-29970), Staphylococcus epidermidis (ATCC-12228), Staphylococcus saprophyticus (ATCC-15305), Streptococcus pneumoniae (ATCC-49619), Streptococcus agalactiae (ATCC-13813), Streptococcus pyogenes (ATCC-19615), Enterococcus faecalis (ATCC-49149), K. pneumonia (ATCC-13883), Proteus mirabilis (ATCC-35659), Pseudomonas aeruginosa (ATCC-27853), Escherichia coli (ATCC-25922), Bacillus cereus (ATCC-14579), Salmonella enterica (ATCC-14028), and Listeria monocytogenes (ATCC-7644) were purchased from BIOBW (China). Eight AMR S. aureus strains were acquired from the College of Biomedicine and Health, Huazhong Agricultural University, China. Culture-based biochemical and susceptibility tests, whole-genome sequencing (WGS), and PCR-Sanger sequencing were used to identify all bacterial strains. Following the manufacturer's directions, genomic DNA (gDNA) was isolated from bacterial cells collected from the bacterial culture medium using a Bacteria Genomic DNA Extraction Kit (TIANamp Biotechnology Co., Ltd., Beijing, China). All extracted gDNA was quantified using a NanoDrop2000 spectrophotometer (Thermo Fisher Scientific UK, no longer available) and kept at –20°C until testing.

### Design of target areas, primers, and probes.

In this study, the thermostable nuclease (*nuc*) gene, which was regarded as specific gene of S. aureus (8, 10, 12), was chosen for identification of S. aureus, and nine distinct AMR genes from four different antibiotic families were for the analysis of antimicrobial resistance, as shown in Fig. 1. The *nuc* gene and AMR gene sequences were obtained from the Antibiotic Resistance Genes Database (https://ardb.cbcb.umd.edu) and the National Center for Biotechnology Information (NCBI, https://www.ncbi.nlm.nih.gov). All WGS of bacteria were obtained from NCBI. The Basic Local Alignment Search Tool (BLAST, https://blast.ncbi.nlm.nih.gov/Blast.cgi) was used to find the most conserved region. Serious RPA primers and probe sets were designed using Primer Premier 5.0 software (Premier Biosoft International, CA, USA) and Primer3Plus (https://www.primer3plus.com/) according to the TwistAmp Assay Design Manual (https://www.twistdx.co.uk/support/rpa-assay-design/). Detailed information is shown in Table 1. Our design included the RPA-FSM version and RPA-LFD version. For the RPA-FSM version, the Exo probe (46 nt) included a flanking 6-carboxyfluorescein deoxythymidine (6-FAM-dT), a corresponding Black Hole Quencher-1 deoxythymidine (BHQ1-dT) quencher group, a tetrahydrofuran (THF) spacer replacing at nt 31 and an adjacent downstream oligonucleotide (15 nt) carrying a C3 spacer (polymerase extension blocking group) at its 3′ end. The internal labels used in the probe are available only on thymines with fewer than approximately 5 intervening nucleotides (nt). For the RPA-LFD type, the reverse primer was tagged with fluorescein isothiocyanate (FITC). The Nfo probes (46 nt) include a 5′-biotin label, a THF spacer replacing at nt 31 and an adjacent downstream oligonucleotide (15 nt) carrying a C3-spacer at its 3′ end. All primers and probes were tested for cross-reactivity using OligoAnalyzer 3.1 from Integrated DNA Technologies, Inc. (https://www.idtdna.com/calc/analyzer). DNA oligonucleotides were synthesized by GENEWIZ Biotechnology Co., Ltd. (Suzhou, China).

### Screening of potential primers and probes.

The RPA using the TwistAmp Basic kit (TwistDX, Cambridge, UK) combined with a 2% AGE assay was used to screen the good primer pairs. According to the suggestions in the TwistAmp Assay Design Manual, the screening strategy was used as follows: By screening all reverse primers against a single forward primer, selecting the best reverse primer, and then using it to screen all forward primers, excellent primer pairs may be obtained in 16 to 20 reactions. Then, the RPA-FSM assay was used to evaluate the best probe and primer combination.

### RPA-FSM assay.

The TwistAmp exo kit (TwistDX Ltd., Maidenhead, UK) was used for the assay in accordance with the manufacturer's instructions. All reaction mixtures were prepared in a final volume of 50 μL containing 2 μL template, 2.1 μL each primer (10 μM), 0.6 μL Exo-probe (10 μM), and other standard reaction components. To commence the reaction, 2.5 μL of magnesium acetate (280 mM) was added and well mixed. Amplification was visualized with a Real Time PCR Detection System (product code: SLAN-96S, Shanghai Hongshi Medical Technology Co., Ltd., Shanghai, China), running for 20 min at 37°C. Fluorescence data were collected once every 30 s for 20 min.

### RPA-LFD assay.

A TwistAmp nfo kit was used to conduct the experiment (TwistDx Ltd., Maidenhead, UK). A total of 2 μL of template, 2.1 μL of each primer (10 mM), 0.6 μL of nfo probe (10 mM), and other standard reaction ingredients were included in each 50 μL reaction mixture. Then, 2.5 μL of magnesium acetate (280 mM) was added and well mixed to start the reaction. After that, the reaction mixture was incubated for 30 min at 37°C. The amplified outcome was diluted to 1/50 and spotted on a commercial LFD (Beijing Baoying Tonghui Biotechnology Co., Ltd., Beijing, China). According to Fig. 1, the commercial LFD was made up of a sample pad, a gold-labeled antibody pad (soaked in mouse-derived AuNP-labeled anti-FITC antibody), a test line (T) (coated with streptavidin), a control line (C) (coated with anti-mouse antibody), and an absorption pad, all of which were organized according to the solvent migration pathway. The final results were visually inspected within 2 to 4 min.

### Optimization of the RPA.

To study the influence of crucial elements, the time, temperature, concentration of primer, probe, and magnesium acetate (280 mM) were measured. The gradient temperature, reaction time, and concentration were tuned to produce the ideal RPA reaction conditions. The impacts related to various parameter optimizations were analyzed using RPA-FSM.

### Diagnostic sensitivity of the RPA.

To assess the sensitivity of the RPA, part of the sequence of the above 10 genes was cloned into pGEM-T Easy Vector (GENEWIZ Biotechnology Co., Ltd., Suzhou, China) and different copies of the recombinant plasmid were used as templates for the RPA reaction. Based on the plasmid (3,015 bp) and insert (300 to 400 bp) sizes, the copy number was estimated in accordance with Whelan et al. and subjected to 10-fold dilutions from 1 × 10^0^ to 1 × 10^5^ copies/L to ascertain the precise sensitivity of the RPA-LFD and RPA-FSM assays (53).

### Diagnostic specificity RPA.

The specificity of the designed RPA-FSM and RPA-LFD was evaluated with positive DNA extracted from other bacterial strains, including S. aureus, K. pneumoniae, P. aeruginosa, P. mirabilis, E. coli, S. pneumoniae, B. cereus, S. hominis, S. enterica, and L. monocytogenes.

### Antibiotic susceptibility tests.

Vitek 2 Compact (bioMérieux China, Inc., China) were used to detect antibiotic susceptibility to 15 antibiotics (penicillin, gentamicin, oxacillin, levofloxacin, rifampin, ciprofloxacin, trimethoprim-sulfamethoxazole, clindamycin, erythromycin, vancomycin, macrodantin, linezolid. Following established protocols, the MIC of an antibiotic was calculated. S. aureus ATCC 29213 was used as the quality control strain for the examination of antibiotic susceptibility.

### Clinical evaluation.

The microbiology team of our lab collected and preprocessed a total of 54 clinical samples from patients of the Second Affiliated Hospital of Shantou University Medical College who were thought to be infected with S. aureus (six blood, three throat swabs, 19 urine, and 26 other samples, in that order). Magnetic Swab DNA Kit (Catalog number: 4992448). All samples were analyzed by PCR, RPA-LFD and RPA-FSM. In order to obtain consistent and repeatable results, each test was repeated three times at least.

### Data analysis.

Data were entered using the Excel program (Microsoft Excel, version 2016). The statistical analysis was performed using SPSS 22 (SPSS Inc., Chicago, IL, USA). A *P* value < 0.05 was considered significant. The positive percent agreement (PPA) was calculated as true positives/(true positives + false negatives) × 100%, and the negative percent agreement (NPA) was calculated as true negatives/(true negatives + false positives) ×100%. The accuracy was computed as (true positives + true negatives)/(true positives + false negatives + true negatives + false positives) ×100%.

### Data availability.

Data are accessible from the authors upon request. On reasonable request, the corresponding author will share the information supporting the study's findings.
