# Supplementary material for: A Rapid Antimicrobial Resistance Diagnostic Platform for Staphylococcus aureus Using Recombinase Polymerase Amplification
Source: Microbiol Spectr. 2023 Mar 28;11(2):e04476-22. doi: 10.1128/spectrum.04476-22 (PMC10100846; doi:10.1128/spectrum.04476-22)
Supplement: Supplemental file 1 — Supplemental material. Download spectrum.04476-22-s0001.pdf, PDF file, 0.6 MB [file spectrum.04476-22-s0001.pdf]

Supporting information

Figure S1. There were no cross-reactions when other bacterial such as Klebsiella pneumoniae and Streptococcus Pneumoniae were tested and detected by RPA-AGE (Fig. S1A), RPA-FSM (Fig. S1B) and RPA-LFD (Fig. S1C).

Figure S2. These primers could not produce cross reaction and could only amplify their own target genes.

Table Legends

Table S1. The optimal reaction system of every gene including concentration of primers, probes, MgAo, reaction temperature and reaction time.

Table S2. All results indicated that our AMR diagnostic detection perform approached the performance of PCR, with a consistency rate of 100%

Table S3. The sample of the study included a total of 54 clinical isolates.

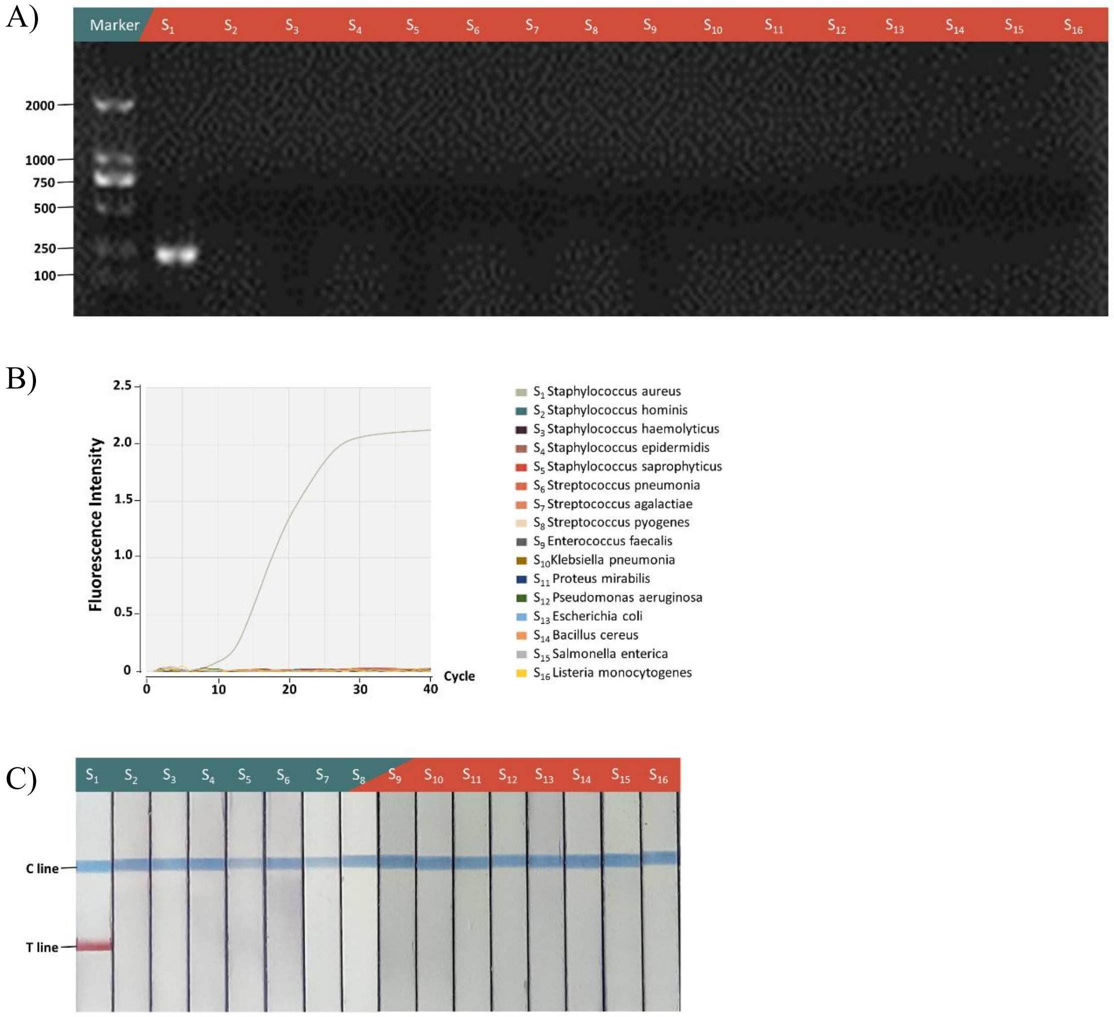

Figure S1. There were no cross-reactions when other bacterial such as Klebsiella pneumoniae and Streptococcus Pneumoniae were tested and detected by RPA-AGE (Fig. S1A), RPA-FSM (Fig. S1B) and RPA-LFD (Fig. S1C).

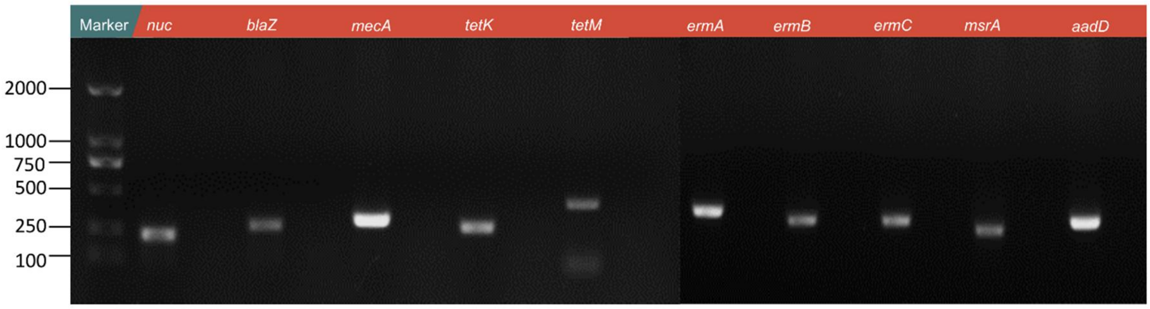

Figure S2. These primers could not produce cross reaction and could only amplify their own target genes.

Table S1. The optimal reaction system of every gene including concentration of primers, probes, MgAo , reaction temperature and reaction time.

A) The best reaction condition for RPA-FSM

genotype of probe	genotype of template	Reaction Temperature (℃)			Reaction Time (min)				
		37	39	42	5	10	15	20	25
nuc-exo	*nuc*	1	5	3	1	3	4	5	5
blaZ-exo	*blaZ*	2	5	4	0	2	4	5	5
mecA-exo	*mecA*	1	5	2	0	3	4	5	5
ermA-exo	*ermA*	3	5	4	1	3	5	5	5
ermB-exo	*ermB*	3	5	3	0	2	4	5	5
ermC-exo	*ermC*	1	5	2	0	3	5	5	5
msrA-exo	*msrA*	3	5	4	1	3	5	5	5
tetK-exo	*tetK*	3	5	2	0	3	5	5	5
tetM-exo	*tetM*	1	5	2	1	3	5	5	5
aadD-exo	*aadD*	3	5	3	0	3	5	5	5

B) The best reaction condition for RPA-LFD

genotype of probe	genotype of template	Reaction Temperature (℃)			Reaction Time (min)				
		37	39	42	5	10	15	20	25
nuc-nfo	*nuc*								
blaZ-nfo	*blaZ*								
mecA-nfo	*mecA*								
ermA-nfo	*ermA*								
ermB-nfo	*ermB*								
ermC-nfo	*ermC*								
msrA-nfo	*msrA*								
tetK-nfo	*tetK*								
tetM-nfo	*tetM*								
aadD-nfo	*aadD*								

Table S2. All results indicated that our AMR diagnostic detection perform approached the performance of PCR, with a consistency rate of 100%

**Clinical isolates**	***nuc***			***blaZ***			***mecA***			***ermA***			***ermB***			***ermC***			***msrA***			***tetK***			***tetM***			***aadD***		
	**PCR**	**RPA-FSM**	**RPA-LFD**	**PCR**	**RPA-FSM**	**RPA-LFD**	**PCR**	**RPA-FSM**	**RPA-LFD**	**PCR**	**RPA-FSM**	**RPA-LFD**	**PCR**	**RPA-FSM**	**RPA-LFD**	**PCR**	**RPA-FSM**	**RPA-LFD**	**PCR**	**RPA-FSM**	**RPA-LFD**	**PCR**	**RPA-FSM**	**RPA-LFD**	**PCR**	**RPA-FSM**	**RPA-LFD**	**PCR**	**RPA-FSM**	**RPA-LFD**
**1**	+	**15.6**	+	+	**14.66**	+	-	**Noct**	-	-	**Noct**	-	-	**Noct**	-	-	**Noct**	-	-	**Noct**	-	+	**11.37**	+	-	**Noct**	-	-	**Noct**	-
**2**	+	**8.35**	+	+	**16.25**	+	+	**12.12**	+	-	**Noct**	-	-	**Noct**	-	+	**7.37**	+	+	**9.55**	+	+	**17.71**	+	-	**Noct**	-	-	**Noct**	-
**3**	+	**14.77**	+	+	**20.52**	+	+	**10.09**	+	-	**Noct**	-	-	**Noct**	-	-	**Noct**	-	-	**Noct**	-	+	**8.9**	+	-	**Noct**	-	-	**Noct**	-
**4**	+	**13.94**	+	+	**19.2**	+	+	**15.58**	+	-	**Noct**	-	-	**Noct**	-	+	**7.54**	+	-	**Noct**	-	-	**Noct**	-	-	**Noct**	-	-	**Noct**	-
**5**	+	**14.58**	+	+	**15.75**	+	-	**Noct**	-	-	**Noct**	-	-	**Noct**	-	-	**Noct**	-	-	**Noct**	-	-	**Noct**	-	-	**Noct**	-	-	**Noct**	-
**6**	+	**6.08**	+	+	**19.22**	+	-	**Noct**	-	-	**Noct**	-	-	**Noct**	-	+	**11.56**	+	-	**Noct**	-	-	**Noct**	-	-	**Noct**	-	-	**Noct**	-
**7**	+	**13.14**	+	+	**8.43**	+	-	**Noct**	-	-	**Noct**	-	-	**Noct**	-	-	**Noct**	-	-	**Noct**	-	+	**15.17**	+	-	**Noct**	-	-	**Noct**	-
**8**	+	**11.78**	+	+	**16.79**	+	-	**Noct**	-	-	**Noct**	-	-	**Noct**	-	+	**6.74**	+	-	**Noct**	-	-	**Noct**	-	-	**Noct**	-	-	**Noct**	-
**9**	+	**15.94**	+	+	**19.68**	+	+	**14.97**	+	-	**Noct**	-	-	**Noct**	-	+	**9.86**	+	-	**Noct**	-	+	**6.53**	+	-	**Noct**	-	+	**20.72**	+
**10**	+	**13.31**	+	+	**10.63**	+	+	**9.34**	+	-	**Noct**	-	-	**Noct**	-	-	**Noct**	-	-	**Noct**	-	-	**Noct**	-	-	**Noct**	-	-	**Noct**	-
**11**	+	**10.31**	+	+	**15.28**	+	-	**Noct**	-	-	**Noct**	-	+	**13.77**	+	-	**Noct**	-	-	**Noct**	-	-	**Noct**	-	+	**12.9**	+	+	**18.69**	+
**12**	+	**12.53**	+	+	**8.52**	+	-	**Noct**	-	-	**Noct**	-	-	**Noct**	-	-	**Noct**	-	-	**Noct**	-	-	**Noct**	-	-	**Noct**	-	-	**Noct**	-
**13**	+	**11.92**	+	+	**20.91**	+	+	**8.38**	+	-	**Noct**	-	-	**Noct**	-	-	**Noct**	-	-	**Noct**	-	-	**Noct**	-	-	**Noct**	-	-	**Noct**	-
**14**	+	**9.28**	+	+	**12.41**	+	+	**7.29**	+	-	**Noct**	-	+	**9.47**	+	-	**Noct**	-	-	**Noct**	-	-	**Noct**	-	-	**Noct**	-	-	**Noct**	-
**15**	+	**9.06**	+	+	**12.48**	+	+	**13**	+	-	**Noct**	-	-	**Noct**	-	-	**Noct**	-	-	**Noct**	-	+	**12.07**	+	-	**Noct**	-	-	**Noct**	-
**16**	+	**16.64**	+	-	**Noct**	-	-	**Noct**	-	+	**15.81**	+	-	**Noct**	-	-	**Noct**	-	-	**Noct**	-	-	**Noct**	-	-	**Noct**	-	-	**Noct**	-
**17**	+	**7.3**	+	-	**Noct**	-	+	**15.45**	+	-	**Noct**	-	+	**18.27**	+	+	**10.07**	+	-	**Noct**	-	+	**6.27**	+	-	**Noct**	-	-	**Noct**	-
**18**	+	**8.14**	+	+	**14.01**	+	-	**Noct**	-	-	**Noct**	-	-	**Noct**	-	-	**Noct**	-	-	**Noct**	-	-	**Noct**	-	-	**Noct**	-	-	**Noct**	-
**19**	+	**14.95**	+	+	**16.35**	+	+	**14.83**	+	-	**Noct**	-	+	**11.02**	+	-	**Noct**	-	-	**Noct**	-	-	**Noct**	-	-	**Noct**	-	-	**Noct**	-
**20**	+	**9.05**	+	+	**15.6**	+	-	**Noct**	-	-	**Noct**	-	+	**12.41**	+	-	**Noct**	-	-	**Noct**	-	-	**Noct**	-	-	**Noct**	-	+	**14.02**	+

21	+	8.85	+	+	14.32	+	-	Noct	-	-	Noct	-	-	Noct	-	-	Noct	-	-	Noct	-	-	Noct	-	-	Noct	-	-	Noct	-
22	+	16.11	+	-	Noct	-	-	Noct	-	-	Noct	-	-	Noct	-	-	Noct	-	-	Noct	-	-	Noct	-	-	Noct	-	-	Noct	-
23	+	6.66	+	+	10.89	+	+	12.83	+	-	Noct	-	+	10.79	+	-	Noct	-	-	Noct	-	-	Noct	-	-	Noct	-	-	Noct	-
24	+	15.38	+	+	8.75	+	-	Noct	-	-	Noct	-	-	Noct	-	-	Noct	-	-	Noct	-	-	Noct	-	-	Noct	-	-	Noct	-
25	+	8.48	+	+	8.44	+	+	9.07	+	-	Noct	-	-	Noct	-	+	13.44	+	-	Noct	-	-	Noct	-	-	Noct	-	-	Noct	-
26	+	13.96	+	+	19.74	+	+	14.67	+	-	Noct	-	+	11.78	+	-	Noct	-	-	Noct	-	+	8.34	+	-	Noct	-	-	Noct	-
27	+	9.94	+	+	11.92	+	-	Noct	-	-	Noct	-	-	Noct	-	-	Noct	-	-	Noct	-	+	6.48	+	-	Noct	-	-	Noct	-
28	+	17.3	+	+	8.11	+	-	Noct	-	-	Noct	-	-	Noct	-	+	9.35	+	-	Noct	-	+	9.29	+	-	Noct	-	-	Noct	-
29	+	11.37	+	+	20.78	+	-	Noct	-	-	Noct	-	-	Noct	-	-	Noct	-	-	Noct	-	-	Noct	-	-	Noct	-	-	Noct	-
30	+	15.88	+	-	Noct	-	-	Noct	-	-	Noct	-	-	Noct	-	-	Noct	-	-	Noct	-	-	Noct	-	-	Noct	-	-	Noct	-
31	+	8.48	+	+	17.94	+	-	Noct	-	+	14.53	+	-	Noct	-	-	Noct	-	-	Noct	-	-	Noct	-	-	Noct	-	-	Noct	-
32	+	17.2	+	+	16.54	+	-	Noct	-	-	Noct	-	+	14.4	+	-	Noct	-	-	Noct	-	-	Noct	-	-	Noct	-	+	13.78	+
33	+	11.13	+	+	20.74	+	-	Noct	-	+	10.04	+	-	Noct	-	-	Noct	-	-	Noct	-	-	Noct	-	-	Noct	-	-	Noct	-
34	+	17.75	+	+	18.34	+	+	12.97	+	-	Noct	-	-	Noct	-	-	Noct	-	-	Noct	-	-	Noct	-	-	Noct	-	-	Noct	-
35	+	8.34	+	-	Noct	-	-	Noct	-	-	Noct	-	-	Noct	-	-	Noct	-	-	Noct	-	-	Noct	-	-	Noct	-	-	Noct	-
36	+	8.79	+	+	18.71	+	+	7.5	+	-	Noct	-	-	Noct	-	-	Noct	-	-	Noct	-	-	Noct	-	-	Noct	-	-	Noct	-
37	+	7.88	+	+	10.9	+	-	Noct	-	-	Noct	-	-	Noct	-	-	Noct	-	-	Noct	-	-	Noct	-	-	Noct	-	-	Noct	-
38	+	13.57	+	-	Noct	-	+	10.54	+	-	Noct	-	-	Noct	-	+	9.68	+	-	Noct	-	-	Noct	-	-	Noct	-	-	Noct	-
39	+	14.52	+	+	16.57	+	+	16.3	+	-	Noct	-	+	9.5	+	-	Noct	-	-	Noct	-	-	Noct	-	-	Noct	-	-	Noct	-
40	+	10.19	+	+	9.46	+	-	Noct	-	-	Noct	-	-	Noct	-	+	10.62	+	-	Noct	-	-	Noct	-	+	10.98	+	+	12.89	+
41	+	8.71	+	+	8.21	+	-	Noct	-	-	Noct	-	-	Noct	-	-	Noct	-	-	Noct	-	+	6.43	+	-	Noct	-	-	Noct	-
42	+	14.36	+	+	11.74	+	-	Noct	-	-	Noct	-	-	Noct	-	-	Noct	-	-	Noct	-	+	8.14	+	-	Noct	-	-	Noct	-

**43**	+	**15.27**	+	+	**15.42**	+	-	**Noct**	-	-	**Noct**	-	-	**Noct**	-	-	**Noct**	-	-	**Noct**	-	+	**7.58**	+	-	**Noct**	-	-	**Noct**	-
**44**	+	**16.05**	+	+	**10.13**	+	+	**10.63**	+	-	**Noct**	-	-	**Noct**	-	-	**Noct**	-	-	**Noct**	-	-	**Noct**	-	-	**Noct**	-	-	**Noct**	-
**45**	+	**7.27**	+	+	**15.53**	+	+	**9.15**	+	-	**Noct**	-	-	**Noct**	-	-	**Noct**	-	-	**Noct**	-	-	**Noct**	-	-	**Noct**	-	-	**Noct**	-
**46**	+	**6.42**	+	-	**Noct**	-	-	**Noct**	-	-	**Noct**	-	-	**Noct**	-	-	**Noct**	-	-	**Noct**	-	+	**12.7**	+	-	**Noct**	-	-	**Noct**	-
**47**	+	**8.2**	+	+	**7.98**	+	-	**Noct**	-	-	**Noct**	-	+	**12.49**	+	+	**16.7**	+	-	**Noct**	-	-	**Noct**	-	-	**Noct**	-	-	**Noct**	-
**48**	+	**14.02**	+	-	**Noct**	-	-	**Noct**	-	-	**Noct**	-	-	**Noct**	-	-	**Noct**	-	-	**Noct**	-	-	**Noct**	-	-	**Noct**	-	-	**Noct**	-
**49**	+	**15.75**	+	-	**Noct**	-	-	**Noct**	-	-	**Noct**	-	-	**Noct**	-	-	**Noct**	-	-	**Noct**	-	-	**Noct**	-	-	**Noct**	-	-	**Noct**	-
**50**	+	**9.85**	+	+	**18.17**	+	+	**16.79**	+	-	**Noct**	-	-	**Noct**	-	+	**9.99**	+	-	**Noct**	-	-	**Noct**	-	-	**Noct**	-	-	**Noct**	-
**51**	+	**6.92**	+	+	**13.84**	+	+	**10.41**	+	-	**Noct**	-	+	**10.3**	+	-	**Noct**	-	-	**Noct**	-	-	**Noct**	-	-	**Noct**	-	+	**14.58**	+
**52**	+	**16.86**	+	+	**20.33**	+	+	**6.73**	+	-	**Noct**	-	-	**Noct**	-	+	**10.56**	+	-	**Noct**	-	-	**Noct**	-	-	**Noct**	-	-	**Noct**	-
**53**	+	**6.59**	+	+	**18.58**	+	-	**Noct**	-	-	**Noct**	-	+	**14.13**	+	-	**Noct**	-	-	**Noct**	-	+	**10.86**	+	+	**10.12**	+	-	**Noct**	-
**54**	+	**6.49**	+	-	**Noct**	-	+	**8.79**	+	-	**Noct**	-	+	**11.13**	+	-	**Noct**	-	-	**Noct**	-	-	**Noct**	-	-	**Noct**	-	-	**Noct**	-

Table S3. The sample of the study included a total of 54 clinical isolates.

Code	Sample Type	*nuc*	Penicillin	*blaZ*	Screening of cefoxitin	Oxacillin	*mecA*	Erythromycin	Clindamycin	Induced clindamycin resistance	*ermA*	*ermB*	*ermC*	*msrA*	Tetracycline	*tetK*	*tetM*	Gentamicin	*aadD*
**1**	**WS**	+	**R**	+	-	**S**	-	**S**	**S**	-	-	-	-	-	**R**	+	-	**S**	-
**2**	**Urine**	+	**R**	+	+	**R**	+	**R**	**R**	+	-	-	+	+	**S**	+	-	**S**	-
**3**	**NGJ**	+	**R**	+	+	**R**	+	**S**	**S**	-	-	-	-	-	**R**	+	-	**S**	-
**4**	**PRE**	+	**R**	+	+	**R**	+	**R**	**R**	+	-	-	+	-	**R**	-	-	**S**	-
**5**	**Urine**	+	**R**	+	-	**S**	-	**S**	**S**	-	-	-	-	-	**S**	-	-	**S**	-
**6**	**CVCT**	+	**R**	+	-	**S**	-	**R**	**R**	+	-	-	+	-	**S**	-	-	**S**	-
**7**	**Urine**	+	**R**	+	-	**S**	-	**S**	**R**	-	-	-	-	-	**R**	+	-	**S**	-
**8**	**Urine**	+	**R**	+	-	**S**	-	**R**	**R**	+	-	-	+	-	**S**	-	-	**S**	-
**9**	**Urine**	+	**R**	+	-	**S**	+	**R**	**R**	+	-	-	+	-	**R**	+	-	**S**	+
**10**	**Pus**	+	**R**	+	+	**R**	+	**S**	**S**	-	-	-	-	-	**S**	-	-	**S**	-
**11**	**PTE**	+	**R**	+	/	**S**	-	**R**	**S**	-	-	+	-	-	**R**	-	+	**S**	+
**12**	**Urine**	+	**R**	+	-	**S**	-	**S**	**S**	-	-	-	-	-	**S**	-	-	**S**	-
**13**	**UVCT**	+	**R**	+	+	**R**	+	**S**	**S**	-	-	-	-	-	**S**	-	-	**S**	-
**14**	**Blood**	+	**R**	+	+	**R**	+	**R**	**R**	-	-	+	-	-	**S**	-	-	**S**	-
**15**	**Urine**	+	**R**	+	+	**R**	+	**S**	**S**	-	-	-	-	-	**R**	+	-	**S**	-
**16**	**BALF**	+	**S**	-	-	**S**	-	**R**	**R**	+	+	-	-	-	**S**	-	-	**S**	-
**17**	**BALF**	+	**R**	-	+	**R**	+	**R**	**R**	-	-	+	+	-	**R**	+	-	**S**	-
**18**	**Urine**	+	**S**	+	-	**S**	-	**S**	**S**	-	-	-	-	-	**S**	-	-	**S**	-
**19**	**Sputum**	+	**R**	+	+	**R**	+	**R**	**R**	-	-	+	-	-	**S**	-	-	**S**	-
**20**	**Blood**	+	**R**	+	-	**S**	-	**R**	**R**	-	-	+	-	-	**S**	-	-	**R**	+
**21**	**UCT**	+	**R**	+	-	**S**	-	**S**	**S**	-	-	-	-	-	**S**	-	-	**S**	-
**22**	**Urine**	+	**S**	-	/	**S**	-	**S**	**S**	-	-	-	-	-	**S**	-	-	**S**	-
**23**	**PRE**	+	**R**	+	+	**R**	+	**R**	**R**	+	-	+	-	-	**S**	-	-	**S**	-
**24**	**Urine**	+	**R**	+	-	**S**	-	**S**	**S**	-	-	-	-	-	**S**	-	-	**S**	-
**25**	**Blood**	+	**R**	+	+	**R**	+	**S**	**R**	-	-	-	+	-	**R**	-	-	**S**	-
**26**	**GTT**	+	**R**	+	+	**R**	+	**R**	**R**	-	-	+	-	-	**R**	+	-	**S**	-
**27**	**Urine**	+	**R**	+	-	**S**	-	**S**	**S**	-	-	-	-	-	**R**	+	-	**S**	-
**28**	**Urine**	+	**R**	+	-	**S**	-	**R**	**R**	-	-	-	+	-	**R**	+	-	**S**	-
**29**	**Sputum**	+	**R**	+	-	**S**	-	**S**	**S**	-	-	-	-	-	**S**	-	-	**S**	-
**30**	**MEE**	+	**S**	-	-	**S**	-	**S**	**S**	-	-	-	-	-	**S**	-	-	**S**	-
**31**	**Urine**	+	**R**	+	-	**S**	-	**R**	**R**	+	+	-	-	-	**S**	-	-	**S**	-
**32**	**CSF**	+	**R**	+	-	/	-	**R**	**R**	-	-	+	-	-	**S**	-	-	**I**	+
**33**	**Urine**	+	**R**	+	-	**S**	-	**R**	**R**	+	+	-	-	-	**S**	-	-	**S**	-
**34**	**CSF**	+	**R**	+	+	**R**	+	**S**	**S**	-	-	-	-	-	**S**	-	-	**S**	-
**35**	**Stool**	+	**R**	-	-	**S**	-	**S**	**S**	-	-	-	-	-	**S**	-	-	**S**	-
**36**	**TS**	+	**R**	+	+	**R**	+	**S**	**S**	-	-	-	-	-	**S**	-	-	**S**	-
**37**	**Urine**	+	**R**	+	-	**S**	-	**S**	**S**	-	-	-	-	-	**S**	-	-	**S**	-
**38**	**GTT**	+	**S**	-	-	**S**	+	**R**	**R**	+	-	-	+	-	**S**	-	-	**S**	-
**39**	**TS**	+	**R**	+	+	**R**	+	**R**	**R**	-	-	+	-	-	**S**	-	-	**S**	-
**40**	**NGJ**	+	**R**	+	+	**R**	-	**R**	**R**	-	-	-	+	-	**R**	-	+	**R**	+
**41**	**Blood**	+	**R**	+	-	**S**	-	**S**	**S**	-	-	-	-	-	**R**	+	-	**S**	-
**42**	**ETT**	+	**R**	+	-	**S**	-	**S**	**S**	-	-	-	-	-	**R**	+	-	**S**	-
**43**	**Urine**	+	**R**	+	-	**S**	-	**S**	**S**	-	-	-	-	-	**R**	+	-	**S**	-
**44**	**Pus**	+	**R**	+	+	**R**	+	**S**	**S**	+	-	-	-	-	**S**	-	-	**S**	-

**45**	**TS**	+	**R**	+	+	**R**	+	**S**	**S**	-	-	-	-	-	**S**	-	-	**S**	-
**46**	**PTE**	+	**S**	-	-	**S**	-	**S**	**S**	-	-	-	-	-	**R**	+	-	**S**	-
**47**	**ETT**	+	**R**	+	-	**S**	-	**R**	**R**	+	-	+	+	-	**S**	-	-	**S**	-
**48**	**MEE**	+	**R**	-	-	**S**	-	**S**	**S**	-	-	-	-	-	**S**	-	-	**S**	-
**49**	**Blood**	+	**S**	-	-	**S**	-	**S**	**S**	-	-	-	-	-	**S**	-	-	**S**	-
**50**	**CVCT**	+	**R**	+	+	**R**	+	**R**	**R**	+	-	-	+	-	**S**	-	-	**S**	-
**51**	**Urine**	+	**R**	+	+	**R**	+	**R**	**R**	-	-	+	-	-	**S**	-	-	**R**	+
**52**	**Urine**	+	**R**	+	+	**R**	+	**R**	**R**	+	-	-	+	-	**S**	-	-	**S**	-
**53**	**Blood**	+	**R**	+	-	**S**	-	**S**	**S**	-	-	+	-	-	**R**	+	+	**S**	-
**54**	**Urine**	+	**R**	-	+	**R**	+	**R**	**R**	-	-	+	-	-	**S**	-	-	**S**	-

PRE Pleural Effusion, PTE Peritoneal Effusion, MEE Middle Ear Effusion, NGJ Neonatal Gastric Juice, BALF Bronchoalveolar Lavage Fluid, CSF Cerebrospinal Fluid, CVCT Central Venous Catheter Tip, ETT Endotracheal Tube Tip, GTT Gavage Tube Tip, UCT Urethral Catheter Tip, UVCT Umbilical Venous Catheter Tip, TS Throat Swabs, WS Wound Swab
